# Severe Psychopathology and Substance Use Disorder Modify the Association Between Housing Trajectories and Food Security Among Homeless Adults

**DOI:** 10.3389/fnut.2021.608811

**Published:** 2021-05-12

**Authors:** James Lachaud, Cilia Mejia-Lancheros, Michael Liu, Ri Wang, Rosane Nisenbaum, Vicky Stergiopoulos, Stephen W. Hwang, Patricia O'Campo

**Affiliations:** ^1^MAP Centre for Urban Health Solutions, Li Ka Shing Knowledge Institute, St. Michael's Hospital, Toronto, ON, Canada; ^2^Harvard Medical School, Boston, MA, United States; ^3^Applied Health Research Centre, Li Ka Shing Knowledge Institute, St. Michael's Hospital, Toronto, ON, Canada; ^4^Dalla Lana School of Public Health, University of Toronto, Toronto, ON, Canada; ^5^Centre for Addiction and Mental Health, Toronto, ON, Canada; ^6^Department of Psychiatry, University of Toronto, Toronto, ON, Canada; ^7^Department of Medicine, University of Toronto, Toronto, ON, Canada

**Keywords:** psychopathology, substance use disorder, housing trajectories, food security, homeless adults

## Abstract

**Purpose:** We examined the housing trajectories of homeless people with mental illness over a follow-up period of 6 years and the association of these trajectories with food security. We then examined the modifying role of psychopathology and alcohol and substance use disorders in this association.

**Materials and Methods:** We followed 487 homeless adults with mental illness at the Toronto site of the At Home/Chez-Soi project—a randomized trial of Housing First. Food security data were collected seven times during the follow-up period. Psychopathology (Colorado Symptom Index score) and alcohol and substance use disorders were assessed at baseline. Housing trajectories were identified using group-based trajectory modeling. Logistic regression was used to estimate the association between housing trajectory groups and food security.

**Results:** Three housing trajectory groups were identified: rapid move to consistent stable housing (34.7%), slow and inconsistent housing (52.1%), and never moved to stable housing (13.2%). Individuals included in the rapid move to consistent housing trajectory group had higher odds of remaining food secure compared with those in the never moved to stable housing trajectory group over the follow-up period [AOR 2.9, 95% CI: 1.3–6.6, *P*-value: 0.009]. However, when interactions were considered, this association was significant among those with moderate psychopathology but not severe psychopathology. Individuals with substance use disorder and in the never moved to stable housing group had the lowest food security status.

**Discussion:** Severe psychopathology and substance use disorders modified the association between housing trajectories and food security.

International Standard Randomized Control Trial Number Register (ISRCTN42520374).

## Introduction

Homelessness (living without stable, safe, permanent, appropriate housing or the immediate prospect, means and ability of acquiring it (1) is detrimental for almost every life dimension of affected individuals. Being homeless is a crucial stress factor for well-being as it reflects severe material deprivation and, in addition, erodes mental, cognitive, and physical health (2–8); exposes individuals to risky conditions and behaviors [e.g., violence, bad meteorological conditions, discrimination; (9, 10)]; and precludes access to such basic services as sanitation, healthcare, water, or food (11–13).

Access to food is a consistent unmet need of homeless populations. Compared with the general population, homeless individuals have lower food security (14), lower nutrient intake, and diet insufficiency (15, 16). Homeless individuals typically cannot access kitchens to prepare meals and preserve perishable foods (17, 18), forcing them to depend on fast food, shelter or community meals, and even food waste to meet their needs or to remain hungry (19–21). The intertwinement of homelessness with mental illness and substance use worsens access to food because it generates competing needs for purchasing medications and substances (22–25).

Housing First (HF) interventions—generally including rent supplements and mental health support services—have been implemented internationally to facilitate exit from homelessness to stable housing while also providing social and health services to support housing stability and enhance health and well-being (26, 27). Studies in Canada and the United States demonstrate the effectiveness of HF interventions in promoting rapid exit from homelessness (28–30). However, two main concerns have been raised about these interventions. First, exiting homelessness remains a complex and non-linear process (31–33). Although some individuals who participate in HF interventions move rapidly and remain stably housed, others are either less successful in remaining stably housed or are never able to become stably housed (31, 32). These different housing trajectories affect and reflect other aspects of the lives of homeless adults. A study by Kerman et al. finds that housing trajectories shape patterns of social services use, independent of whether individuals are in the intervention or standard treatment arms (34). For example, participants who achieved sustained housing stability across both intervention and standard treatment groups have similar patterns of emergency department use, hospitalization, inpatient psychiatric admission, or food bank use. Within the intervention group, social and health services use was largely different among subgroups of individuals with different housing patterns.

Second, as for several mental health outcomes, HF interventions seem to have limited effect on quality of life of homeless individuals, including the satisfaction of some basic needs, such as food security, which is at the physiological level of Maslow's pyramid of needs and well-being (35–37). Previous analysis of HF in five Canadian cities over a 2-year period showed that access to stable housing was not sufficient to improve food security among individuals with mental illness (37). However, the heterogeneity of housing trajectories was not adequately accounted for in that study.

Here, we examine patterns of exiting homelessness to stable housing (housing trajectory groups) among homeless individuals with mental illness participating in the Toronto At Home/Chez Soi Study over a follow-up period of 6 years (38). This long follow-up period provided enough time to observe consistent changes over time, contrary to previous analyses that were limited to 2 years (31, 33, 39). Second, we ask whether these housing trajectory groups were associated with food security of study participants over the study period. Finally, we investigated whether the association between housing trajectory groups and food security differed across the severity of psychopathology and alcohol and substance use disorders. We hypothesized that a rapid move to stable housing was associated with consistent food security over the follow-up period and that this association was weaker among participants with severe psychopathology and alcohol and substance use disorders.

## Materials and Methods

### AH/CS Intervention

This study used data from the Toronto site of the At Home/Chez Soi (AH/CS) study, which was a randomized trial that compared the HF intervention (provision of mental health support services, such as assertive community treatment or intensive case management, plus rent supplement) to treatment as usual (TAU) (access to social, housing, and health services available in the community) (40). Participants were recruited from Toronto community agencies, shelters, clinics, and directly from the street between October 2009 and July 2011. The Toronto AH/CS participants were initially followed for 2 years (Phase I) between October 2009 and July 2013. In 2013, the study received additional funding to extend its follow-up period from January 2014 to March 2017 (Phase II) for a total follow-up period of approximately 6 years. Detailed information on study recruitment, design, population, and measurement instruments is reported elsewhere (41).

Four inclusion criteria were used to select AH/CS study participants: (1) being 18 years of age or older, (2) being absolutely homeless or precariously housed, (3) having a diagnosed severe mental disorder, and (4) not being served by assertive community treatment or an intensive case management program. Prior to randomization, participants were stratified by their level of needs for mental health services as high needs (HN) and moderate needs (MN). The level of need for mental health services was assessed using a combined algorithm that included having a psychotic disorder or bipolar affective disorder with psychotic symptoms [based on the Mini International Neuropsychiatric Interview 6.0 (MINI)], low community functioning [based on the Multnomah Community Ability Scale (MCAS)], presence of a comorbid substance use disorder, and prior history of hospitalizations and incarcerations (40–43). Out of the 575 Toronto participants, 197 participants met criteria to be classified as HN and 378 as MN. Then, according to their level of need, participants were randomly assigned to either the HF treatment or TAU. Participants assigned to the treatment group with HN received HF support services with assertive community treatment (ACT), and those with MN received HF support services with intensive case management (ICM) treatment. Participants assigned to the TAU group continued to have access to housing and social and health support services locally available in their communities.

### Ethical considerations

The Toronto AH/CS study received approval from the St. Michael's Hospital Research Ethics Board (Canada), and all participants gave informed written consent to participate in the AH/CS study. The AH/CS study is also registered with the International Standard Randomized Control Trial Number Register (ISRCTN42520374).

### Measures and Operational Definitions

#### Stable Housing

We captured stable housing through a residential timeline follow-back calendar (RTLFB) questionnaire (31, 44), which was administered every 3 months (Phase I) or 6 months (Phase II) to track the number of days living/sleeping in different types of housing accommodations. An accommodation was defined to be stable housing if the participant had tenancy rights or was expected to remain in the same accommodation for more than 6 months. For each follow-up year, participants were classified as being stably housed if they remained in stable housing for at least 75% of RTLFB-accounted days over a calendar year.

#### Consistent Food Security

The modified version of the U.S. Adult Food Security Survey Module (US FSSM) [U.S. Department of Agriculture, Economic Service Research (45) U.S. Adult Food Security Survey Module: Three-Stage Design, with Screeners, 2012] was used to assess the food security status of each participant over the 30 days prior to interview time points. Data were gathered every 6 months during phase I and every 12 months during phase II up to seven times across the 6-year follow-up period. This instrument has been validated in previous studies to assess food security for individuals experiencing homelessness (46–48). It contains 10 items related to food access, and the code responses were summed up to compute the food security score. This score ranges from 0 to 10 and classifies food security status into two main groups: food secure [those with high food security (score = 0) and marginal food security (score = 1–2)] and food insecure [those with low food security (score = 3–5), and very low food security (score = 6–10)] (37, 49, 50).

Participants were classified as being consistently food secure over the follow-up time if they were in the “food secure” group for more than 50% of the duration of their follow-up interviews. For example, a participant with four follow-up interviews had to be food secure at least three times (more than 50%) to be classified as consistently food secure. To ensure that participants had a minimum follow-up number of interviews for the analysis, we excluded those who had fewer than three food security interviews (*n* = 88).

#### Covariates

##### HF Intervention

Because the present study is embedded within an HF intervention, we considered the indicator of HF treatment (HF vs. TAU) as a covariate to adjust for the housing trajectory groups over the follow-up period.

Other covariates included sociodemographic variables (age at baseline (in years), self-reported gender (classified as male or not male), ethno-racial group membership [ethno-racial and not ethno-racial group), and marital status (single or not)], number of children under 18 years, and lifetime duration of homelessness prior to study enrolment (<3 and ≥3 years). No other gender categories were considered because there were fewer than 10 non-binary individuals in our sample. Participants were asked whether they used a food bank in the last 6 months prior to food security interviews, and we counted the number of times over the follow-up period.

### Modifier Variables

#### Severe psychopathology

We used the Colorado Symptom Index score (CSI) at baseline to assess for the effect of psychopathology on food security (40, 51). CSI is a widely used self-report measure of psychiatric symptomatology and includes 14 items. Participants were asked how often they experienced specific psychiatric symptoms, such as “How often have you felt nervous, tense, worried, frustrated, or afraid?” or “How often have your voices, thoughts, or feelings interfered with your doing things?” Their responses were graded using a five-point Likert-items rated from “not at all” (1) to “at least every day” (5). Total scores ranged from 14 to 70, and higher scores indicated more severe psychiatric symptoms. Previous studies show that this index has high internal consistency (Cronbach's alpha = 0.92) (52, 53). CSI was dichotomized using a preestablished clinical threshold 30 or higher to indicate individuals with high (1) or low (0) psychopathology severity (52).

#### Alcohol and Substance disorders

Alcohol and non-alcohol substance use disorders were identified separately based on DSM-IV criteria using the MINI 6.0 and were evaluated at the time participants were screened for entering the study (40, 54).

### Statistical Methods

We used group-based trajectory modeling to identify patterns of exiting homelessness to stable housing over the 6-year follow-up period (55). This modeling technique allows for the identification of clusters of individuals who followed a similar housing trajectory over time (56). Assuming a logistic distribution of housing stability, it uses intercept and time as change parameters to estimate latent trajectory groups. For the shape of trajectory groups, we tested different polynomial growth factors (linear, quadratic, and cubic time factors) and determined the optimal number of trajectory groups through the Bayesian information criterion (BIC). To determine the best-fit trajectory shapes, we used the average posterior probability measure and the weighted odds of correct classification (OCC) (55). Afterward, the model was adjusted for HF intervention group membership. All models were estimated using the module *Traj* in Stata 15 (55).

Next, we used logistic regression models to estimate odds ratios and 95% confidence intervals for the association between housing trajectory groups and consistent food security over the 6-year follow-up period. Then, we adjusted the model including the following covariates: age, self-reported gender, ethno-racial group membership, marital status, number of children under 18 years, lifetime duration of homelessness, food bank use, severe psychopathology, alcohol use disorder, and substance use disorder. To assess the modifying effect of severe psychopathology and alcohol and substance use disorders, we reestimated the model with interaction terms, and the interaction graphs are presented. All statistical analyses were performed with Stata version 15 (StataCorp. 2017. Stata Statistical Software: Release 15. College Station, TX: StataCorp LLC.).

## Results

Sample characteristics are summarized in Table 1. Of 487 participants, 39.8% were consistently food secure (with high or marginal food security status) over the study period. Severe psychopathology was present in 77.6%, and 43.9 and 47.4% had alcohol and substance use disorders, respectively.

**Table 1:** Characteristics of the At Home/Chez Soi participants at baseline (*n* = 487).

**Variable**	***n***	**%**
**Consistently food secure**		
Yes	194	39.8
No	293	60.2
**Gender**		
Male	333	68.4
Female	154	31.6
**Age group**		
18–34	172	35.3
35–44	127	26.1
45–74	188	38.6
**Education level**		
Middle/high school	228	48.1
Completed high school	85	17.9
Graduate/post-graduate	161	34.0
**Ethno-racial group membership**		
Ethno-racial	282	57.9
not ethno-racial	205	42.1
**Marital status**		
Not married	327	67.1
Married	160	32.9
**Lifetime duration of homelessness**		
<3 years	210	44.97
3 years or more	257	55.03
Number of children under 18 [Mean (SD)]	487	1.6 (1.1)
Food bank use [Mean (SD)]	487	1.97 (1.96)
**Intervention**		
Housing First (HF)	272	55.9
Treatment as usual (TAU)	215	44.1
Severe psychopathology (CSI*≥30)	378	77.6
Alcohol use disorder	214	43.9
Substance use disorder	231	47.4

* *Colorado symptoms index—SD, standard deviation*.

### Housing Trajectories

**Figure 1:**
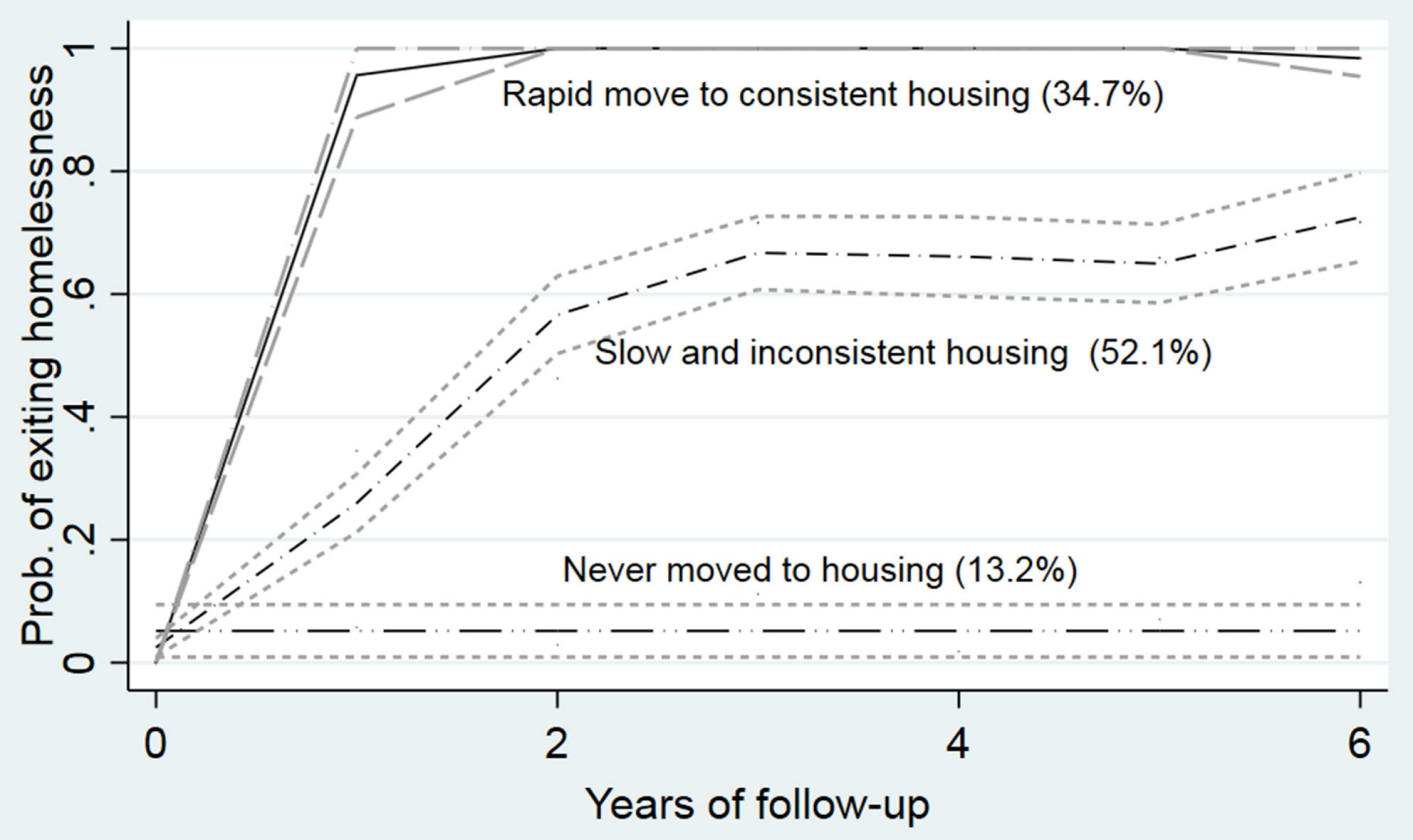
Housing trajectory groups over time points with 95% confidence interval.

Three housing trajectory groups were identified: a rapid move to consistent stable housing trajectory with a quadratic form, a slow and inconsistent housing trajectory with a cubic form, and a never moved to stable housing trajectory (see Table 2 and Figure 1). Of the 487 participants, 34.7% of participants were classified in the rapid move to consistent stable housing group and 52.1% in the slow and inconsistent housing group; 13.2% never moved to stable housing during the study period. The BIC fit statistics confirmed this model as the best fit model (BIC for the two-group model = −1416.08; BIC_3_ = −1400.10, and BIC_4_ = −1455.53). The average posterior probability (>0.70) and the OCC-weighted posterior portability (>5) also indicate good fit. The adjusted model demonstrates that the HF intervention influences the trajectories, mainly by increasing the probability of having a rapid move to consistent housing and decreasing the probability of never moving or slow compared with the slow and inconsistent housing trajectory. Average posterior probabilities and two of the OCC-weighted posterior probabilities indicate improvement after the adjustment.

**Table 2:** Housing Trajectory Groups adjusted from Group-Based Trajectory modeling.

**Parameters**	**Model I**				**Model adjusted for intervention group**					
	**Intercept**	**Linear**	**Quadratic**	**Cubic**	**Intercept**	**Linear**	**Quadratic**	**Cubic**	**AH**	***P*-value**
Rapid to consistent housing	−12.2	16.4	−2.3		−14.9	20.9	−3.0		**2.1**	**0.001**
Slow and inconsistent housing	−3.5	3.2	−0.8	0.1	−3.7	3.4	−0.9	0.1	**Ref**.	
Never moved to stable housing	−3.0				−2.9				**−1.2**	**0.017**
BIC	−1400.1				−1353.6					
**Group**	***N*** **(%)**	**Group APP**		**OCC weighted**	***N*** **(%)**		**Group APP**	**OCC weighted**		
**Membership and posterior probability**										
Rapid to consistent housing	206 (38.4)	0.88		11.29	186 (34.7)		0.92	22.64		
Slow and inconsistent housing	264 (49.2)	0.86		6.54	279 (52.1)		0.86	5.69		
Never moved to stable housing	66 (12.4)	0.83		34.16	71 (13.2)		0.86	39.22		

*APP, average of the maximum posterior probability of assignments. OCC, Odds of correct classification weighted posterior probability. Bold: significant at a level of 5%*.

### Housing Trajectory and Consistent Food Security

Compared with those in the never moved to stable housing trajectory, those in the rapid move to consistent housing stability group were more likely to be consistently food secure, AOR 2.9 [95% CI (1.3–6.6)] (see Table 3). Likewise, those with severe psychopathology and a substance use disorder were less likely to be consistently food secure (AOR 0.39 [95% CI (0.24–0.63)]) and 41% (AOR 0.59 [95% CI (0.37–0.95)]), respectively.

**Table 3:** Multivariable logistic regressions for consistent food security and housing trajectory groups adjusted for baseline characteristics.

**Variable**	**AOR (95% CI)**	***P*-value**
**Housing Trajectory groups**		
Never moved to stable housing (ref.)		
Slow and inconsistent housing	2.2 (1.0–4.8)	0.053
Rapid and stable housing	2.9 (1.3–6.6)	**0.009**
Severe psychopathology	0.39 (0.24–0.63)	**0.001**
Alcohol use disorder	1.01 (0.63–1.61)	0.964
Substance use disorder	0.59 (0.37–0.95)	**0.031**
Intercept	1.24 (0.40–3.90)	0.709

*AOR, adjusted odd ratios*.

*The model is adjusted for the following variables: Gender, age, education level, ethno-racial group, marital status, lifetime homelessness, number of children under 18, and food bank use. Bold: significant at a level of 5%*.

### Modification Effects of Psychopathology and Substance Use Disorder

**Figure 2:**
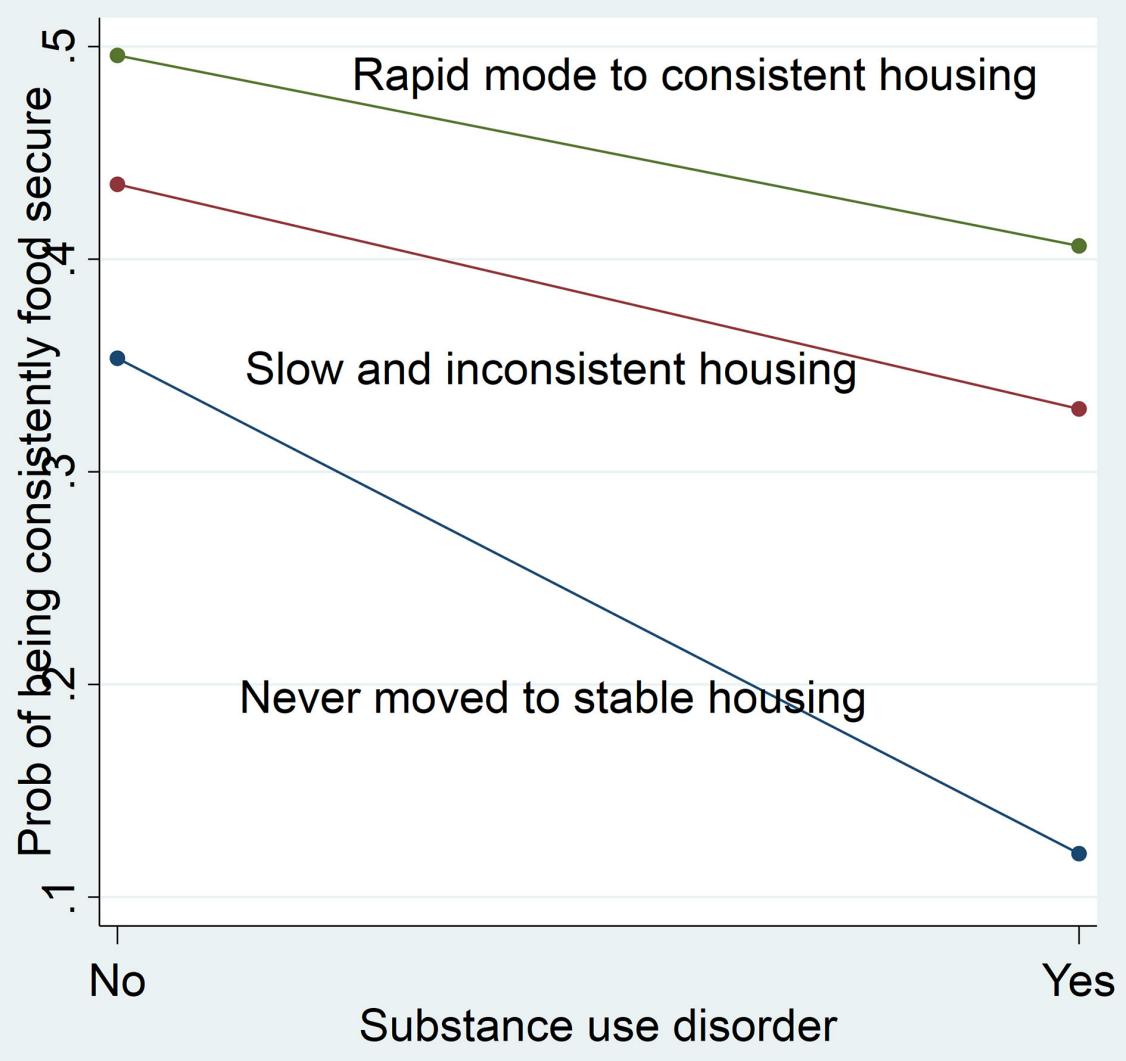
Psychopathology modifies the effect of housing trajectory on food security.

**Figure 3:**
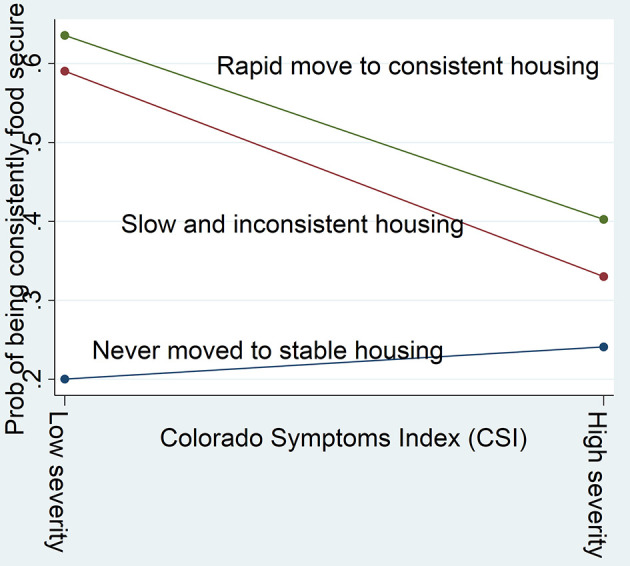
Substance use disorders modify the effect of housing trajectory on food security.

As shown in Figure 2, among participants with low psychopathology, those who moved to housing (rapid move to consistent housing and slow and inconsistent housing) were 40% more likely to be consistently food secure compared with those who never moved to stable housing. Conversely, among participants with severe psychopathology, no difference is observed between the housing trajectory groups. Likewise, those with no alcohol or substance use disorders and who never moved to stable housing were least likely to be consistently food secure (Figure 3). No modification effect was observed for alcohol use disorder.

## Discussion

This study examined housing trajectories and their association with food security among homeless adults with mental illness in a large urban center. Three housing trajectory groups were identified: a rapid move to consistent housing group, a slow and inconsistent housing group, and a never moved to stable housing group. These results corroborate findings from previous studies on the complexity of exiting homelessness. Adair et al., using data from the multisite at Home/Chez Soi study of HF, found different housing trajectories over a 2-year period, including a group of almost no time housed (29%); a group of rapid and sustained housing (33%); and several small groups, such as slow and sustained housing, early housing, and gradually lost, rapid gain, and steep decline (31). Another study among homeless youth in Los Angeles County identified three trajectories over a 2-year period, a consistently sheltered group, a group with a high probability of finding and maintaining shelter over time, and a last group who remained inconsistently sheltered over a long term (33). Contrary to these studies conducted over a period up to 2 years, our study looked at pathways out of homeless to housing over a period of 6 years.

Our results indicate that providing housing is only one step toward supporting people experiencing homelessness by helping them leave streets and shelters and entering a residence. However, even after achieving housing, these individuals face several personal, economic, and social challenges and extreme poverty that can impede their achievement of long-term housing stability (32, 57–59). Moving into stable housing is accompanied by additional living costs (e.g., paying for utilities) that compete with other basic needs (e.g., food), rendering individuals vulnerable to eviction and repeated homelessness.

Our results also show that individuals in the rapid move to consistent housing group were more likely to be food secure compared with those in the never moved to stable housing group over the follow-up period. However, this association disappeared for those with severe psychopathology. Furthermore, those with substance use disorders and in the never moved to stable housing group were least likely to be food secure. Our results support findings from a recent study conducted by O'Campo et al. (37) on the role of housing stability as a key determinant of food security. Our findings offer new insight into the complexity of how pathways out of homelessness interact with mental health and substance use disorders to prevent individuals from achieving food security. The combination of a difficult housing trajectory with mental illness might prevent employment, forcing individuals to make trade-offs between food security, stable housing (e.g., rent), and other basic needs (59–61).

There are several study limitations to note. First, food security data may be affected by recall bias, which might influence the accuracy of reported findings. Second, there were not enough follow-up interviews to consistently assess food security over the entire study period. More food security interviews over the same follow-up period would have required reducing the time interval between interviews. Finally, this study focuses on homeless adults who have mental illness and is also limited to an only site, Toronto City, and cannot be generalized to all homeless adults.

Notwithstanding these limitations, this study provides a comprehensive view of the complexity of exiting homelessness to stable housing and its influence on food security. The analysis also offers insight into how psychopathology and substance use disorders contribute to poor food security even after homeless people achieve long-term housing stability. This study has two main policy implications. First, housing interventions must focus on achieving housing stability over a long period of time with special attention on factors that increase risk for unsuccessful housing trajectories. Second, it is important to enhance housing interventions with food security programs to better address the food needs of homeless adults with severe psychopathology and substance use disorders.

## Data Availability Statement

Data cannot be made publicly available for both ethical and legal reasons. They were collected from randomized trial implemented within a hospital setting, St. Michael's Hospital in Toronto, which conferred the participants the status of patient. Data also contain information related to mental health status of the participants. Data collection, use, and disclosure are governed by the Personal Health Information Protection Act (PHIPA, 2004) and must not be disclosed without their written informed consent, as was stated in the written informed consent form by law. As the study addresses a specific and small subpopulation, any combination of three to four variables can facilitate the identification of some participants. Nonetheless, Home/Chez Soi Toronto Data will be available to investigators for studies that have received approval from research ethics boards. Study proposals and data access requests should be sent to Evie Gogosis at evie.gogosis@unityhealth.to.

## Ethics Statement

This study was conducted according to the guidelines laid down in the Declaration of Helsinki and all procedures involving research study participants were approved by the Research Ethics Board of St. Michael's Hospital (Canada). Written informed consent was obtained from all subjects/patients. The At Home/Chez-Soi study is registered with the International Standard Randomized Control Trial Number Register (ISRCTN42520374). The patients/participants provided their written informed consent to participate in this study.

## Author Contributions

JL, CM-L, and PO'C conceived and designed the present research study. JL conducted the final statistical analysis, interpreted the data, and wrote the first manuscript draft. CM-L, RW, RN, and ML assisted in the study design, interpretation of the results, and revision of the first manuscript draft. PO'C, VS, and SH were also the principal investigators of the AH/CS study, Toronto site. All the co-authors revised and approved the present final manuscript version. All authors contributed to the interpretation of the results and critical revision and edition of the final manuscript.

## Conflict of Interest

The authors declare that the research was conducted in the absence of any commercial or financial relationships that could be construed as a potential conflict of interest.

## References

[1] Gaetz S Barr C Friesen A Harris B Hill C Kovacs-Burns K. Canadian Definition of Homelessness. Available online at: https://www.homelesshub.ca/sites/default/files/COHhomelessdefinition.pdf (accessed December 30, 2020).

[2] Childress S Reitzel LR Maria DS Kendzor DE Moisiuc A Businelle MS. Mental Illness and substance use problems in relation to homelessness onset. Am J Health Behav. (2015) 39:549–55. 10.5993/AJHB.39.4.11 26018103

[3] Davidson D Chrosniak LD Wanschura P Flinn JM. Indications of reduced prefrontal cortical function in chronically homeless adults. Commun Mental Health J. (2014) 50:548–52. 10.1007/s10597-013-9664-9 24337470

[4] Fazel S Khosla V Doll H Geddes J. The prevalence of mental disorders among the homeless in Western countries: systematic review and meta-regression analysis. PLoS Med. (2008) 5:1670–81. 10.1371/journal.pmed.0050225 19053169 PMC2592351

[5] Fazel S Geddes JR Kushel M. The health of homeless people in high-income countries: Descriptive epidemiology, health consequences, and clinical and policy recommendations. Lancet. (2014) 384:1529–40. 10.1016/S0140-6736(14)61132-6 25390578 PMC4520328

[6] Hwang SW. Is homelessness hazardous to your health? Can J Public Health. (2002) 93:407–10. 10.1007/BF03405026 12448860 PMC6980210

[7] Pluck G Lee KH David R Macleod DC Spence SA Parks RW. Neurobehavioural and cognitive function is linked to childhood trauma in homeless adults. Br J Clin Psychol. (2011) 50:33–45. 10.1348/014466510X490253 21332519

[8] Romaszko J Cymes I Dragańska E Kuchta R Glińska-Lewczuk K. Mortality among the homeless: causes and meteorological relationships. PLoS ONE. (2017) 12:e0189938. 10.1371/journal.pone.0189938 29267330 PMC5739436

[9] Gaetz S. Safe streets for whom? Homeless youth, social exclusion, and criminal victimization. Can J Criminol Crim Just. (2004) 46:423–56. 10.3138/cjccj.46.4.423

[10] Skosireva A O'Campo P Zerger S Chambers C Gapka S Stergiopoulos V. Perceived discrimination among homeless adults with mental illness in health care settings. BMC Health Serv Res. (2014) 14:376. 10.1186/1472-6963-14-376 25196184 PMC4176588

[11] Baggett TP O'Connell JJ Singer DE Rigotti NA. The unmet health care needs of homeless adults: a national study. Am J Public Health. (2010) 100:1326–33. 10.2105/AJPH.2009.180109 20466953 PMC2882397

[12] Gozdzik A Salehi R O'Campo P Stergiopoulos V Hwang SW. Cardiovascular risk factors and 30-year cardiovascular risk in homeless. BMC Public Health. (2015) 15:165. 10.1186/s12889-015-1472-4 25886157 PMC4339633

[13] Khandor E Mason K Chambers C Rossiter K Cowan L Hwang SW. Access to primary health care among homeless adults in Toronto, Canada: results from the Street Health survey. Open Med. (2011) 5, e94–e103. 21915240 PMC3148004

[14] Baggett TP Singer DE Rao SR O'Connell JJ Bharel M Rigotti NA. Food insufficiency and health services utilization in a national sample of homeless adults. J Gen Int Med. (2011) 26, 627–34. 10.1007/s11606-011-1638-4 21279455 PMC3101971

[15] Tarasuk V Dachner N Poland B Gaetz S. Food deprivation is integral to the ‘hand to mouth' existence of homeless youths in Toronto. Public Health Nutrition. (2009) 12:1437–42. 10.1017/S1368980008004291 19144218

[16] Tarasuk V Dachner N Li J. Homeless youth in Toronto are nutritionally vulnerable. J Nutrition. (2018) 135:1926–33. 10.1093/jn/135.8.1926 16046718

[17] Herault N Ribar DC. Food insecurity and homelessness in the Journeys Home survey. J Hous Econ. (2017) 37:52–66. 10.1016/j.jhe.2017.05.001

[18] O'Flaherty B. Individual homelessness: entries, exits, and policy. J Hous Econ. (2012) 21:77–100. 10.1016/j.jhe.2012.04.006

[19] Geller L. Putting Housing First. Canadian Nurse (2014). Avcailable online at: https://www.canadian-nurse.com/articles/issues/2014/june-2014/putting-housing-first (accessed September 30, 2020). 25076576

[20] Parpouchi M Moniruzzaman A Rezansoff SN Russolillo A Somers JM. Characteristics of adherence to methadone maintenance treatment over a 15-year period among homeless adults experiencing mental illness. Addict Behav Rep. (2017) 6:106–11. 10.1016/j.abrep.2017.09.001 29450244 PMC5800549

[21] Rezansoff SN Moniruzzaman A Fazel S Procyshyn R Somers JM. Adherence to antipsychotic medication among homeless adults in Vancouver, Canada: a 15-year retrospective cohort study. Soc Psychiatry Psychiatr Epidemiol. (2016) 51:1623–32. 10.1007/s00127-016-1259-7 27338740 PMC5091737

[22] Baer TE Scherer EA Fleegler EW Hassan A. Food insecurity and the burden of health-related social problems in an urban youth population. J Adolesc Health. (2015) 57:601–7. 10.1016/j.jadohealth.2015.08.013 26592328

[23] Jessiman-Perreault G McIntyre L. The household food insecurity gradient and potential reductions in adverse population mental health outcomes in Canadian adults. SSM Populat Health. (2017) 3:464–72. 10.1016/J.SSMPH.2017.05.013 29349239 PMC5769073

[24] Martin MS Maddocks E Chen Y Gilman SE Colman I. Food insecurity and mental illness: disproportionate impacts in the context of perceived stress and social isolation. Public Health. (2016) 132:86–91. 10.1016/j.puhe.2015.11.014 26795678

[25] Tarasuk V Mitchell A Dachner N. Household Food Insecurity in Canada 2014. Toronto, ON: Research to identify policy options to reduce food insecurity (PROOF) (2016).

[26] Goering PN Streiner DL. Putting housing first: the evidence and impact. Can J Psychiatry. (2015) 60:465–6. 10.1177/070674371506001101 26720503 PMC4679126

[27] Tsemberis S Gulcur L Nakae M. Housing first, consumer choice, and harm reduction for homeless individuals with a dual diagnosis. Am J Public Health. (2004) 94:651–6. 10.2105/AJPH.94.4.651 15054020 PMC1448313

[28] Aubry T Goering P Veldhuizen S Adair CE Bourque J Distasio J. A multiple-city RCT of housing first with assertive community treatment for homeless canadians with serious mental illness. Psychiatr Serv. (2016) 67:275–81. 10.1176/appi.ps.201400587 26620289

[29] O'Campo P Stergiopoulos V Nir P Levy M Misir V Chum A. How did a Housing First intervention improve health and social outcomes among homeless adults with mental illness in Toronto? Two-year outcomes from a randomised trial. BMJ Open. (2016) 6:e010581. 10.1136/bmjopen-2015-010581 27619826 PMC5030577

[30] Stergiopoulos V Hwang SW Gozdzik A Nisenbaum R Latimer E Rabouin D. Effect of scattered-site housing using rent supplements and intensive case management on housing stability among homeless adults with mental illness: a randomized trial. J Am Med Assoc. (2015) 313:905–15. 10.1001/jama.2015.1163 25734732

[31] Adair CE Streiner DL Barnhart R Kopp B Veldhuizen S Patterson M. Outcome trajectories among homeless individuals with mental disorders in a multisite randomised controlled trial of housing first. Can J Psychiatry. (2017) 62:30–9. 10.1177/0706743716645302 27310238 PMC5302104

[32] Patterson ML Moniruzzaman A Somers JM. History of foster care among homeless adults with mental illness in Vancouver, British Columbia: a precursor to trajectories of risk. BMC Psychiatry. (2015) 15:32. 10.1186/s12888-015-0411-3 25884810 PMC4349718

[33] Tevendale HD Comulada WS Lightfoot MA. Finding shelter: two-year housing trajectories among homeless youth. J Adolesc Health. (2011) 49:615–20. 10.1016/j.jadohealth.2011.04.021 22098772 PMC10480485

[34] Kerman N Sylvestre J Aubry T Distasio J. The effects of housing stability on service use among homeless adults with mental illness in a randomized controlled trial of housing first. BMC Health Serv Res. (2018) 18:190. 10.1186/s12913-018-3028-7 29558927 PMC5859427

[35] Lachaud J Mejia-Lancheros C Wang R Wiens K Nisenbaum R Stergiopoulos V. Mental and substance use disorders and food insecurity among homeless adults participating in the At Home/Chez Soi Study. PLoS ONE. (2020) 15:e0232001. 10.1371/journal.pone.0232001 32324795 PMC7179857

[36] Lester D. Measuring Maslow's Hierarchy of Needs. Psychological Reports. (2013) 113:15–7. 10.2466/02.20.PR0.113x16z1 24340796

[37] O'Campo P Hwang SW Gozdzik A Schuler A Kaufman-Shriqui V Poremski D. Food security among individuals experiencing homelessness and mental illness in the at Home/Chez Soi Trial. Public Health Nutr. (2017) 20:2023–33. 10.1017/S1368980017000489 28560947 PMC10261387

[38] Lachaud J Mejia-Lancheros C Liu M Wang R Nisenbaum R Stergiopoulos V. Severe psychopathology and substance use disorder modify the association between housing trajectories and food security among homeless adults. Res Square [Preprint]. (2020) 1–18. 10.21203/rs.3.rs-26111/v1 PMC8152664 34055849

[39] Aubry T Klodawsky F Coulombe D. Comparing the housing trajectories of different classes within a diverse homeless population. Am J Commun Psychol. (2012) 49:142–55. 10.1007/s10464-011-9444-z 21557093

[40] Goering PN Streiner DL Adair C Aubry T Barker J Distasio J. The at Home/Chez Soi trial protocol: a pragmatic, multi-site, randomised controlled trial of a Housing First intervention for homeless individuals with mental illness in five Canadian cities. BMJ Open. (2011) 1:e000323. 10.1136/bmjopen-2011-000323 22102645 PMC3221290

[41] Hwang SW Stergiopoulos V O'Campo P Gozdzik A. Ending homelessness among people with mental illness: the at Home/Chez Soi randomized trial of a Housing First intervention in Toronto. BMC Public Health. (2012) 12:787. 10.1186/1471-2458-12-787 22978561 PMC3538556

[42] Barker S Barron N McFarland BH Bigelow DA Carnahan T. A community ability scale for chronically mentally Ill consumers: part II. Applications. Commun Mental Health J. (1994) 30:459–72. 10.1007/bf02189063 7851100

[43] van Vliet IM de Beurs. E. [The MINI-International Neuropsychiatric Interview. A brief structured diagnostic psychiatric interview for DSM-IV en ICD-10 psychiatric disorders]. Tijdschr Psychiatr. (2007) 49:393–7. 17614093

[44] Tsemberis S McHugo G Williams V Hanrahan P Srefancic A. Measuring homelessness and residential stability: the residential time-line follow-back inventory. J Commun Psychol. (2007) 35:29–42. 10.1002/jcop.20132

[45] U.S. Department of Agriculture, Economic Service Research. US Adult Food Security Survey Module: Three-Stage Design, with Screeners (2012).

[46] D'andreamatteo C and Slater J. Measuring food security in canadian homeless adult men. Can J Diet Pract Res. (2018) 79:42–5. 10.3148/cjdpr-2017-026 28971686

[47] Gundersen C Engelhard EE Crumbaugh AS Seligman HK. Brief assessment of food insecurity accurately identifies high-risk US adults. Public Health Nutr. (2017) 20:1367–71. 10.1017/S1368980017000180 28215190 PMC10261547

[48] Holland AC Kennedy MC Hwang SW. The assessment of food security in homeless individuals : a comparison of the Food Security Survey Module and the Household Food Insecurity Access Scale. Public Health Nutr. (2011) 14:2254–9. 10.1017/S1368980011001327 21740619

[49] Bickel G Nord M Price C Hamilton W Cook J. Guide to Measuring Household Food Security, Revised 2000. Measuring food security in the United States: reports of the federal interagency food security measurement project. United States Department of Agriculture (2000). Available online at: https://www.fns.usda.gov/guide-measuring-household-food-security-revised-2000 (accessed April 15, 2021).

[50] United States Department of Agriculture Economic Research Security. Definition of Food Security (2018). Available online at: https://www.ers.usda.gov/topics/food-nutrition-assistance/food-security-in-the-us/definitions-of-food-security.aspx (accessed December 30, 2020).

[51] Conrad KJ Yagelka JR Matters MD Rich AR Williams V Buchanan M. Reliability and validity of a modified Colorado Symptom Index in a national homeless sample. Mental Health Serv Res. (2001) 3:141–53. 10.1023/A:1011571531303 11718206

[52] Boothroyd RA Chen HJ. The psychometric properties of the Colorado Symptom Index. Administr Policy Mental Health Mental Health Serv Res. (2008) 35:370–8. 10.1007/s10488-008-0179-6 18561020

[53] Shern DL Wilson NZ Coen AS Patrick DC Foster M Bartsch DA. Client outcomes II: longitudinal client data from the Colorado treatment outcome study. Milbank Q. (1994) 72:123. 10.2307/3350341 8164605

[54] Sheehan D Lecrubier Y Harnett-Sheehan K Janavs J Weiller E Hergueta T. the development and validation of a structured diagnostic psychiatric interview for DSM-IV and ICD-10. J Clin Psychiatry. (1998) 59(Suppl. 20), 22–3. 10.1016/S0924-9338(99)80239-9 9881538

[55] Nagin DS Odgers CL. Group-based trajectory modeling in clinical research. Ann Rev Clin Psychol. (2010) 6:109–38. 10.1146/annurev.clinpsy.121208.131413 20192788

[56] Frankfurt S Frazier P Syed M Jung KR. Using group-based trajectory and growth mixture modeling to identify classes of change trajectories. Counsel Psychol. (2016) 44:622–60. 10.1177/0011000016658097

[57] Nelson G Patterson M Kirst M Macnaughton E Isaak CA Nolin D. Life changes among homeless persons with mental illness: a longitudinal study of housing first and usual treatment. Psychiatr Serv. (2015) 66:592–7. 10.1176/appi.ps.201400201 25686813

[58] Patterson ML Rezansoff S Currie L Somers JM. Trajectories of recovery among homeless adults with mental illness who participated in a randomised controlled trial of Housing First: a longitudinal, narrative analysis. BMJ Open. (2013) 3:e003442. 10.1136/bmjopen-2013-003442 24022392 PMC3773649

[59] Stergiopoulos V Gozdzik A O'Campo P Holtby AR Jeyaratnam J Tsemberis S. Housing First: exploring participants' early support needs. BMC Health Serv Res. (2014) 14:167. 10.1186/1472-6963-14-167 24725374 PMC4021373

[60] Jones AD. Food insecurity and mental health status: a global analysis of 149 countries. Am J Prevent Med. (2017) 53:264–73. 10.1016/j.amepre.2017.04.008 28457747

[61] St-Germain AAF Tarasuk V. High vulnerability to household food insecurity in a sample of Canadian renter households in government-subsidized housing. Can J Public Health. (2017) 108:e129–4. 10.17269/CJPH.108.5879 31820414 PMC6972222

